# Implicit and Explicit Motor Learning Interventions Have Similar Effects on Walking Speed in People After Stroke: A Randomized Controlled Trial

**DOI:** 10.1093/ptj/pzab017

**Published:** 2021-01-22

**Authors:** Li-Juan Jie, Melanie Kleynen, Kenneth Meijer, Anna Beurskens, Susy Braun

**Affiliations:** 1 Research Centre for Nutrition, Lifestyle and Exercise, Zuyd University of Applied Sciences, Heerlen, Netherlands; 2 Department of Family Medicine, CAPHRI School for Public Health and Primary Care, Maastricht University, Maastricht, Netherlands; 3 Department of Nutrition and Movement Sciences, NUTRIM School for Nutrition and Translational Research in Metabolism, Maastricht University, Maastricht, Netherlands; 4 Research Centre for Autonomy and Participation of Persons with a Chronic Illness, Zuyd University of Applied Sciences, Heerlen, Netherlands; 5 Department of Health Services Research, CAPHRI School for Public Health and Primary Care, Maastricht University, Maastricht, Netherlands

**Keywords:** Motor Control and Motor Learning, Implicit Learning, Stroke, Gait: Gait Training, Analogy Learning, Rehabilitation

## Abstract

**Objective:**

Clinicians may use implicit or explicit motor learning approaches to facilitate motor learning of patients with stroke. Implicit motor learning approaches have shown promising results in healthy populations. The purpose of this study was to assess whether an implicit motor learning walking intervention is more effective compared with an explicit motor learning walking intervention delivered at home regarding walking speed in people after stroke in the chronic phase of recovery.

**Methods:**

This randomized, controlled, single-blind trial was conducted in the home environment. The 79 participants, who were in the chronic phase after stroke (age = 66.4 [SD = 11.0] years; time poststroke = 70.1 [SD = 64.3] months; walking speed = 0.7 [SD = 0.3] m/s; Berg Balance Scale score = 44.5 [SD = 9.5]), were randomly assigned to an implicit (n = 38) or explicit (n = 41) group. Analogy learning was used as the implicit motor learning walking intervention, whereas the explicit motor learning walking intervention consisted of detailed verbal instructions. Both groups received 9 training sessions (30 minutes each), for a period of 3 weeks, targeted at improving quality of walking. The primary outcome was walking speed measured by the 10-Meter Walk Test at a comfortable walking pace. Outcomes were assessed at baseline, immediately after intervention, and 1 month postintervention.

**Results:**

No statistically or clinically relevant differences between groups were obtained postintervention (between-group difference was estimated at 0.02 m/s [95% CI = −0.04 to 0.08] and at follow-up (between-group difference estimated at −0.02 m/s [95% CI = −0.09 to 0.05]).

**Conclusion:**

Implicit motor learning was not superior to explicit motor learning to improve walking speed in people after stroke in the chronic phase of recovery.

**Impact:**

To our knowledge, this is the first study to examine the effects of implicit compared with explicit motor learning on a functional task in people after stroke. Results indicate that physical therapists can use (tailored) implicit and explicit motor learning strategies to improve walking speed in people after stroke who are in the chronic phase of recovery.

## Introduction

One of the most practiced motor skills in stroke rehabilitation is walking.^1^ In general, therapists can use implicit or explicit forms of learning to facilitate improvement of gait. Explicit motor learning can be referred to as a more conscious form of learning characterized by the generation of verbal knowledge (ie, facts and rules about movement performance) and involvement of cognitive resources.^2^ In contrast, implicit motor learning is assumed to take place without much knowledge of the underlying facts and rules of motor skills and has been described as “learning that progresses with no or minimal increase in the verbal knowledge of movement performance and without awareness.”^2^^(p2)^ Within current clinical practice, therapists tend to structure therapy in a more explicit manner or switch between implicit and explicit learning approaches.^3–5^ However, this might not always be efficient. For people after stroke, who often experience cognitive impairments,^6^ it can be difficult to process large amounts of verbal explicit information. Implicit motor learning, on the other hand, strives to minimize the involvement of cognitive resources, especially working memory,^7^ and may therefore be more feasible for people after stroke who apart from physical constraints also suffer from cognitive impairments. Studies show that people after stroke are able to learn implicitly and that performance of an implicitly learned task might be more stable under dual-task conditions and more durable over time.^8^ However, there is still a lack of studies comparing the effects of implicit motor learning post-stroke to explicit motor learning within clinically relevant tasks. To be clinically meaningful, implicit and explicit motor learning approaches need to be tailored to the individual needs of the patients and performed in the real-life situations.

One practical approach to induce implicit motor learning is through the use of analogies. In analogy learning, the learner is provided with 1 single metaphor (or analogy) that strives to encompass all underlying (explicit) knowledge that is necessary to complete the motor skill. For example, to facilitate step length, a therapist could provide the analogy “Walk as if you follow the footprints in the sand.”^9^ Although no technical (explicit) instructions are given, the analogy may facilitate for example a more symmetrical gait, the foot strike from heel to toe and foot clearance. Studies in athletes have shown that analogy learning led to better and more stable performance under dual-task conditions.^10^^,^^11^ Within the neurological population, first pilot studies revealed the feasibility of analogy learning and demonstrated its potential as both clinically relevant and statistically significant changes in walking performance could be obtained.^9^^,^^12^^,^^13^ In the current study, the effects of analogy learning were compared with detailed verbal instructions when training the clinically relevant task “walking” in a real-life setting (home environment).

To our knowledge, this is the first randomized controlled trial that examines the effects of implicit motor learning facilitated by analogies compared with explicit motor learning on a functional walking task in people after stroke. Contrary to earlier studies examining implicit motor learning using the same analogy for the entire group,^11^ the current study also tailored the interventions towards the individual needs, preferences, and abilities of the patients. The research question was: Is a 3-week implicit motor learning walking intervention (analogies) more effective compared with a 3-week explicit motor learning walking intervention (verbal detailed instructions) delivered at home with regard to walking speed in people after stroke who are in the chronic phase of recovery? Walking speed was chosen due to its integrated results on other gait parameters, for example, step length,^14^ stability,^15^ and functional outcomes.^16^ It was hypothesized that implicit motor learning would result in greater improvements of walking speed post intervention (especially at 1 month post intervention).

## Methods

### Study Design and Participants

The study adopted a randomized, controlled, single-blinded study design. Full details of the study protocol have been published elsewhere.^17^ Information on this trial was reported in adherence to the CONSORT checklist.^18^ Recruitment of participants took place via community practices, rehabilitation institutes in the region, and a local health-related newspaper. Participants were included if they were >6 months after stroke, had a self-selected walking speed slower than 1.0 m/s, and were able to communicate in Dutch and to complete a 3-stage command. Participants were excluded if they were unable to walk a minimum distance of 10 m, could not ambulate on level surfaces without manual contact with another person (Functional Ambulation Scale < 3), or had additional impairments not related to stroke that significantly influenced their gait pattern (eg, Parkinson disease).

### Randomization and Masking

A randomization list was generated using a web-based randomization program and was only available to an independent researcher not involved in the delivery of the interventions or measurements. Patients were randomly assigned (1:1) to either the implicit or explicit motor learning condition (block size of 4). The assessors were blind to the treatment allocation. The therapists were aware of the treatment condition they provided. Patients were not told which condition they received and were asked to not reveal details about the treatment to the blinded assessors.

### Interventions

**Figure 1:**
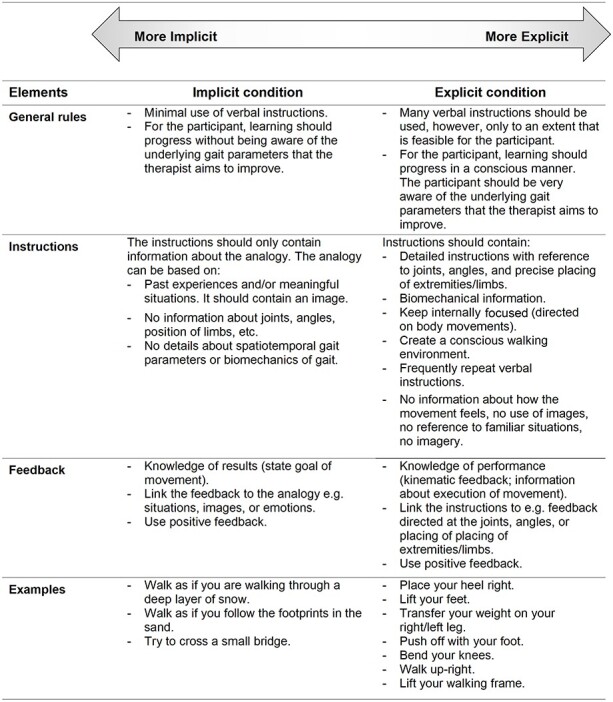
Characteristics of the interventions. Reproduced from Jie et al^17^ under the terms of Creative Commons Attribution 4.0 license.

The interventions aimed to improve quality of walking performance in people after stroke. The to-be-improved gait parameters were chosen according to the analyses of the therapist and the needs of the patients. In total, 9 training sessions were provided over a 3-week-long intervention period. Each session lasted 30 minutes. Within a case-study, this duration and frequency of sessions were sufficient to result in clinically meaningful changes.^13^ An intervention guideline outlining how the implicit and explicit motor learning intervention should be delivered was developed for therapists in the trial. The guideline was developed with physical therapists and client representatives and was based on the previous pilot studies and experiences.^9^^,^^13^^,^^17^ Prior to the trial, 5 standardization training sessions with the therapists took place to discuss and explicate the intervention guideline with example cases. In both interventions, a therapist examined the participant’s walking pattern and defined the underlying gait parameters that could potentially influence walking speed. More details about the interventions and main characteristics with regard to instructions and feedback are described in Figure 1.^17^

#### The Implicit Intervention

The main focus for the implicit intervention was creating a learning situation in which the learner was not (or minimally) aware of the underlying rules of the practiced motor skill. The concept of analogy learning formed the basis to guide the implicit intervention because (1) it has been shown to adopt characteristics of implicit learning^10^ and (2) it offers therapists a practical and feasible tool to apply therapy.^12^^,^^13^ The participants were provided with an analogy that aimed to improve the walking performance and was meaningful to them, for example, pretend that you are walking as if you are following the footprints in the sand.^12^ To create meaningful analogies, the procedure similar to Kleynen et al^13^ was followed together with the participants.

#### The Explicit Intervention

The main focus for the explicit intervention was creating a learning situation in which the learner was very aware of the learning process, for example, in which he/she can precisely explicate the underlying facts and rules that are necessary to perform the motor skill. Therefore, the participant was provided with detailed explicit instructions on their gait performance, for example, “While walking pay attention your foot placement and motion. Place your left foot in front of your right foot. Make sure that you strike the ground with your heel first. Then roll through from heel to toe. Finally push off with your toe.”

### Outcomes

#### Demographic Information

The following demographic information and clinical characteristics were collected: age, gender, time post stroke, affected side, use of walking aids, educational level, cognitive level (Montreal Cognitive Assessment, [MoCA]),^19^ static balance and fall risk (Berg Balance Scale),^20^ mobility disability (Rivermead Mobility Index),^21^ and ability to make movements outside the synergetic patterns (Fügl-Meyer assessment of the lower limb).^22^ To assess the propensity for conscious motor processing, the Dutch version of the Movement Specific Reinvestment Scale^23^^,^^24^ was used.

#### Outcome Assessment

The primary outcome measure was walking speed, which was calculated by measuring the walking time on a 10-Meter Walk Test (10MWT; 10 m/time [s]).^25^ A pathway was marked at 0 (first line) and 10 m (second line). No acceleration or deceleration distances were used. Participants were asked to walk “at a comfortable pace” and start from one end to the other end of the path. They were allowed to use walking aids, but assistance of the assessor did not take place. The assessor stood at the beginning of the pathway and used a stopwatch to measure the time (seconds) over the 10-m distance. The assessor stopped the stopwatch as soon as the participant’s limb crossed the second marker. The 10MWT was performed 3 times, and the average score of the combined walks was used within the study.^26^ Secondary outcomes measures were the modified Dynamic Gait Index,^27^ motor and cognitive Dual Task performance,^28^ Movement Specific Reinvestment Scale adapted for gait,^17^^,^^29^ verbal protocol, Stroke and Aphasia Quality of Life Scale-39,^30^ Global Perceived Effect scale,^31^ and verbal protocol.

#### Assessment of the Dual Task

Both single- and dual-task performance were measured. Single-task performance included only completing the motor (walking) or cognitive (tone-counting task^28^) task. The dual-task performance included walking while simultaneously completing a tone-counting task. Motor dual-task interference was assessed by calculating the Dual Task Effects according to the formula below of Kelly et al.^32^^,^^33^ This led to the following formula for the motor task (walking speed) performance:

$$\begin{align*} & \mathrm{Dual}\ \mathrm{Task}\ \mathrm{Effects}\ \left(\%\right) \\ & =\frac{\left( dual\ task^{\prime } gait\ speed^{\prime }- single\ task^{\prime } gait\ speed^{\prime}\right)}{single\ task^{\prime } gait\ speed^{\prime }}\times 100\% \end{align*}$$

Cognitive dual-task interference was assessed through calculating the error scores (actual minus estimate) and converting these to percentages, as done before by Wilson et al.^28^ The error scores were not yet relative to single task. Therefore, the dual-task error scores were subtracted from the single-task error scores. Both the motor and cognitive task performances were expressed in percentages. Negative percentages indicate that performance deteriorated relative to single task, whereas positive scores indicate relative improvements of the dual-task performance.

#### Verbal Protocol

To assess the amount of explicit knowledge, a verbal protocol questionnaire was administered after the 3-week intervention.^8^ Explicit knowledge was assessed by examining the number of explicit rules that the participant used during walking. More information of the definition of “explicit rule” is described elsewhere.^17^ The answers of the verbal protocol were screened by 2 independent researchers who were blind to the experimental intervention.

### Sample Size Calculation and Statistical Analyses

The sample size calculation resulted in a minimum group size of 33 participants per group. The power was set at beta = .80, the significance level at alpha = .05, and a SD of .23.^34^ The minimal clinically important difference (MCID) of 0.16 m/s for walking speed was set as the minimal change.^35^ Considering 10.0% of participants may be lost during (drop-out) and another 10.0% after the intervention (loss to follow-up), this study aimed to recruit 40 participants per group. The statistical analyses were performed using IBM SPSS (version 24). Baseline characteristics of the 2 groups were reported using frequency distributions and descriptive statistics. For the intention-to-treat analyses, the data of all participants who received the intervention were analyzed according to their original treatment allocation. Treatment effects on numerical data were assessed using a linear mixed model. The model represented group, time, and group × time as fixed factors. For the repeated measures (balanced design), an unstructured covariance structure was used. The linear mixed model analyses uses all available data, corrects for baseline differences, and accounts for dependency of data.

Statistical analyses of the primary outcome were also described in relation to clinically relevant differences between groups (MCID: 0.16 m/s).^35^ In the per-protocol analyses, the data of participants were excluded if they did not receive the intervention as intended, that is, when protocol deviations occurred in 2 or more (of the 9) sessions. Possible protocol deviations were self-reported (subjective) in therapists’ logs, and 10 gait-training sessions were randomly audio-recorded (objective) and evaluated to detect protocol deviations. Furthermore, people who did not meet the inclusion criteria or people who dropped out were excluded in the per-protocol analysis. Descriptive subgroup analysis was performed on cognition to explore whether cognitive abilities (MoCA ≤ 21) might influence the effect of the interventions. The verbal protocol was assessed only once, and an independent *t* test was used to compare results between the groups.

### Role of Funding Source

This work was supported by Nationaal Regieorgaan Praktijkgericht Onderzoek SIA (RAAKPRO; grant number 2014–01-49PRO).

## Results

### Flow of Participants Through the Trial

**Figure 2:**
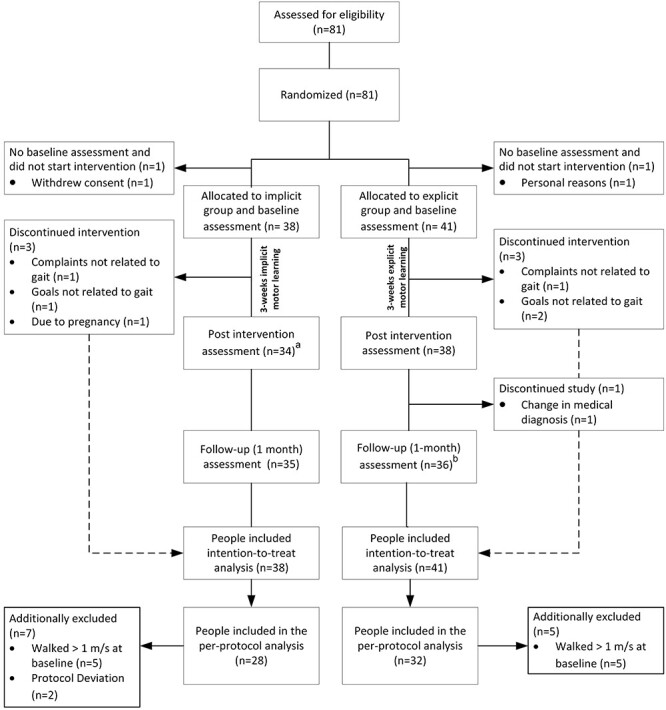
Flowchart of the trial. ^a^One participant was unavailable for the post intervention assessment (n = 1). ^b^One participant was unavailable for the follow-up assessment (n = 1).

The flowchart of the trial is presented in Figure 2. Between May 19, 2017, and September 19, 2018, a total of 81 people were assessed for eligibility and randomized. Two participants (2.5%) did not start with the study. One participant withdrew due to diagnoses with additional impairments that severely influenced his gait. The other participant decided to stop due to personal reasons. All participants (n = 79) who started the intervention were included in the primary intention-to-treat analysis. Demographics and baseline characteristics are presented in Table 1. There were no apparent differences between the groups at baseline.

**Table 1:** Baseline Characteristics of Participants

**Characteristic**	**Implicit (n = 38)**	**Explicit (n = 41)**
General characteristics		
Age (y), mean (SD)	64.6 (9.4)	67.8 (11.6)
Sex, n males (%)	24 (63.2%)	25 (61.0%)
Length (cm), mean (SD)	171.7 (8.0)	172.0 (8.8)
Educational level, no. (%)		
Elementary education	0 (0.0%)	1 (2.4%)
Secondary education	20 (52.6%)	18 (43.9%)
Vocational training	10 (26.3%)	11 (26.8%)
University	8 (21.1%)	11 (26.8%)
Stroke characteristics		
Time poststroke (mo), mean (SD)	72.8 (59.3)	67.5 (69.1)
Side of stroke, no. right (%)	19 (50.0%)	16 (39.0%)
Independent walking, no. (%)		
Walk unaided	9 (23.7%)	9 (22.0%)
Walk with stick	29 (67.3%)	32 (78.1%)
Motor characteristics		
Berg Balance Scale (0–56), mean (SD)	45.5 (11.6)	43.5 (8.9)
Rivermead Mobility Index (0–15), mean (SD)	11.6 (2.5)	11.3 (2.7)
Fügl-Mayer Assessment (0–34), mean (SD)	23.5 (8.0)	22.2 (8.1)*^a^*
Cognitive characteristics		
Montreal Cognitive Assessment (0/30), mean (SD)	24.7 (4.2)	23.2 (6.2)
Conscious motor control preference		
Movement Specific Reinvestment Scale (0–10), mean (SD)	4.9 (2.5)	5.1 (2.6)

^*a*^Due to fatigue, 1 participant from the explicit group was unable to complete the Fügl-Mayer Assessment.

### Compliance With the Trial

Two participants in the explicit group (2.5%) deviated from the protocol with regard to the provided instructions (>2 explicit instructions within the implicit intervention). Analysis revealed that in retrospect, 10 participants (5 participants from the implicit and 5 participants of the explicit group; 12.7%) did not meet the inclusion criteria of walking slower than 1 m/s at baseline. In addition, 3 participants (1 participant of the implicit and 2 participants of the explicit group; 3.8%) wanted to improve overall fitness but had no specific goals related to gait and therefore discontinued with the intervention. Two participants (1 participant in the implicit and 1 participant in the explicit group; 2.5%) stopped due to other complaints not related to gait. Furthermore, the medical diagnosis of 1 participant in the explicit group (initially stroke; 1.3%) was changed during the intervention (misdiagnosed by his medical doctor). Due to pregnancy, another participant in the implicit group dropped out of the intervention (1.3%). All available data of these 19 participants (24.1%) were included in the primary intention-to-treat analysis but were excluded in the per-protocol analysis.

### Results of the Intention-to-Treat Analysis

**Figure 3:**
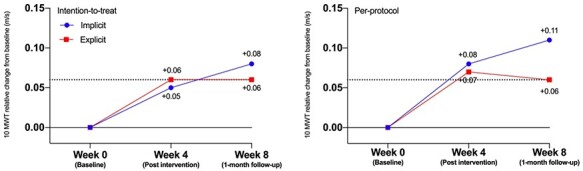
Performance of the 10MWT (10 Meter Walking Test). The dotted lines indicate the meaningful threshold for clinically relevant improvements over time according to Perara et al.^43^

**Figure 4:**
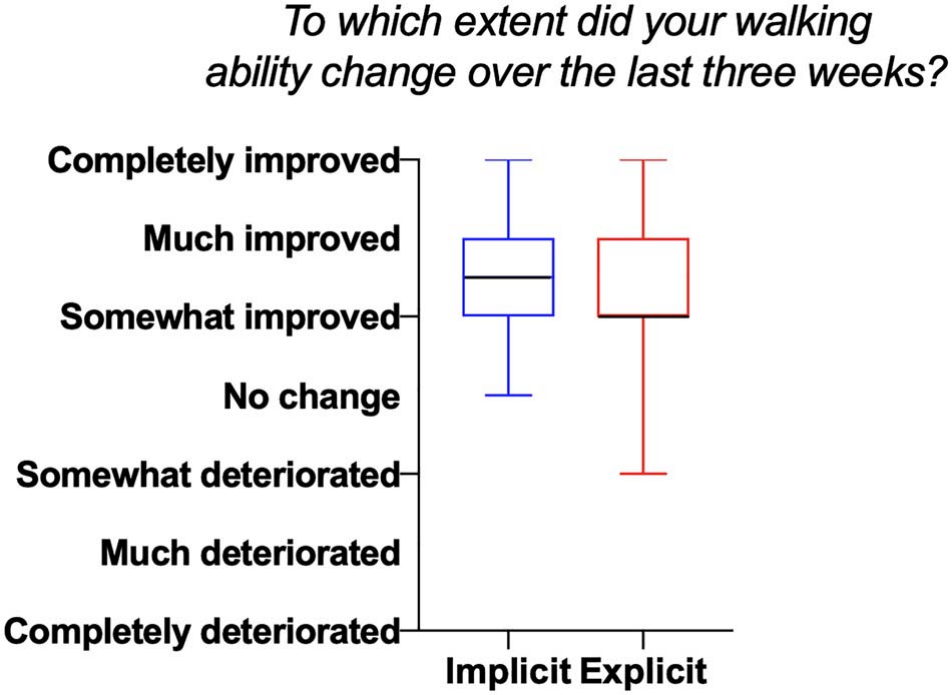
Boxplot of the Global Perceived Effect.

Table 2 presents the observed means (SD) per group and time point, the within-group differences, and the estimated between-group differences of the implicit versus explicit group. Mixed linear models revealed no statistically significant differences between the groups immediately after (difference estimate 0.02 m/s [95% CI = −0.04 to 0.08], *P* = .498) and 1 month post intervention (difference estimate −0.02 m/s [95% CI = −0.09 to 0.05], *P* = .563; see Tab. 2; Fig. 3). Also, no clinically relevant (MCID: 0.16 m/s^31^) differences between groups were observed. No statistically significant differences in favor of any group were obtained on any of the other secondary outcome parameters (Tab. 2). Over time, within groups, positive changes were observed in outcome measures related to gait function (10 Meter Walk Test, Dynamic Gait Index, Dual Task motor) and quality of life (Stroke and Aphasia Quality of Life Scale-39). Participants’ perceived effects regarding the intervention were similar for both groups (see Fig. 4). Regarding the verbal protocol, on average, people in the implicit group accumulated significantly fewer explicit rules (M = 0.38, R = 0 to 2 rules, SE = 0.10) compared with the explicit group (M = 2.42, R = 0 to 6 rules, SE = 0.27; t(68) = −7.07, *P* < .05) after the intervention.

**Table 2:** Mean (SD) of Groups, Mean (SD) Difference Within Groups, and Estimated Mean (95% CI) Difference Between Groups as Established With Linear Mixed Model^a^

	**Groups**						**Within Group Difference** ^***b***^				**Estimated Between Group Difference**	
**Outcome**	**Week 0 (baseline)**		**Week 4 (Post intervention)**		**Week 8 (1 Month Follow-up)**		**Week 4 Minus Week 0**		**Week 8 Minus Week 0**		**Week 4 Minus Week 0**	**Week 8 Minus Week 0**
	**Implicit (n = 38)**	**Explicit (n = 41)**	**Implicit (n = 34)**	**Explicit (n = 38)**	**Implicit (n = 35)**	**Explicit (n = 36)**	**Implicit**	**Explicit**	**Implicit**	**Explicit**	**Implicit-Explicit**	**Implicit-Explicit**
10 MWT (m/s)	0.71 (0.37)	0.70 (0.29)	0.76 (0.37)	0.76 (0.33)	0.79 (0.40)	0.76 (0.34)	0.05 (0.13)	0.07 (0.13)	0.08 (0.15)	0.06 (0.14)	0.02 (−0.04 to 0.08)	−0.02 (−0.09 to 0.05)
DTE motor task (%)	−7.2 (23.3)	−7.7 (38.4)	6.4 (33.9)	3.4 (38.4)	3.4 (34.5)	−6.3 (22.9)	11.2 (29.6)	5.1 (24.6)	9.8 (30.1)	0.6 (24.8)	−2.21 (−17.38 to 12.66)	−10.45 (−22.91 to 2.02)
DTE cognitive task (%)	−6.3 (20.2)	−12.8 (23.6)	−6.3 (21.9)	0.1 (54.8)	−13.6 (52.9)	8.4 (57.4)	1.8 (31.5)	8.5 (51.7)	−5.8 (57.5)	15.9 (51.0)	7.35 (−12.48 to 27.19)	23.57 (−2.04 to 49.17)
mDGI (0–64)	35.9 (15.3)	33.7 (13.2)	39.6 (15.0)	36.5 (14.1)	39.3 (16.4)	36.4 (14.5)	3.5 (5.3)	2.5 (4.6)	3.7 (6.1)	2.5 (5.0)	−0.75 (−2.98 to 1.49)	−1.28 (−3.93 to 1.38)
MSRS (0–10)	4.9 (2.5)	5.1 (2.6)	5.1 (2.6)	4.4 (2.1)	4.9 (2.4)	4.8 (2.6)	0.4 (2.2)	−0.7 (2.5)	0.2 (2.3)	−0.3 (2.4)	−0.84 (−1.74 to 0.07)	0.34 (−1.29 to 0.62)
SAQOL-39 (1–195)	152 (23)	148 (25)	–	–	161 (22)	152 (30)	–	–	10 (15)	5 (17)	–	−5.9 (−13.34 to 1.50)

^*^a^*^10MWT = 10-Meter Walk Test; DTE = Dual Task Effect; mDGI = modified Dynamic Gait Index; SAQOL-39 = Stroke and Aphasia Quality of Life Scale-39.

^*^b^*^Within-group differences were calculated pairwise; missing cases were excluded. Small anomalies in subtraction are due to the effects of rounding.

#### Subgroup Analysis on Cognition

In total, 15 people (implicit group n = 5; explicit group n = 10) had a MoCA score ≤21. No trend in favor of the implicit intervention was observed in the descriptive subgroup analysis on cognition (see Suppl. Tab. 1).

### Results of the Per-Protocol

The per-protocol analyses led to slightly larger changes between groups but again did not lead to statistically significant after (difference estimate −0.06 m/s [95% CI = −0.13 to 0.02], *P* = .140) or clinically relevant effects (MCID: 0.16 m/s) between groups on the primary outcome (see right graph Fig. 3 and Suppl. Tab. 2).

## Discussion

People after stroke in the chronic phase of recovery who received an implicit motor learning walking intervention (analogies) under the guidance of physical therapists in their home environments had similar effects on walking speed compared with those who received an explicit motor learning walking intervention (verbal detailed instructions). Neither statistically nor clinically relevant differences between groups were found as between-group differences (intention to treat ∆ 0.02 m/s; per protocol ∆ 0.05 m/s) did not exceed the chosen clinically relevant threshold of 0.16 m/s.^35^ Similarly, based on the descriptive subgroup analysis on cognition (MoCA ≤ 21), no trend in favor of the implicit intervention was observed (Suppl. Tab. 1). No statistical subgroup analysis on cognition was performed due to small group sizes.

To our knowledge, this was the first and largest trial in the field of stroke rehabilitation to examine the effectiveness of implicit motor learning to improve the functional “walking” task within a clinically relevant context (home environment of the patient).^36^ The results of this study did not replicate the more promising findings on implicit motor learning in stroke from earlier studies, generally performed in more standardized laboratory settings and/or with nonfunctional tasks, for example.^37–39^ A variety of factors related to the selection of participants (selection bias), use of the 10MWT as primary outcome measure (information bias), and operationalization of the intervention (contrasts) may have influenced the results and led to these neutral findings.

First, a selection bias may have occurred. To increase generalizability of the results and gain a better insight into the potential effects in clinical practice, we chose to include a sample of stroke patients, which reflects the heterogeneity of the stroke population as seen in rehabilitation. The researched target population group therefore showed a large variability in terms of demographics as well as physical and cognitive abilities. This heterogeneity may reflect reality in practice but might also have diminished the results. Further, the erroneous inclusion of 10 participants whose baseline walking speed exceeded the inclusion criterion may have led to a ceiling effect. This ceiling effect might explain the larger, but not significant, trend towards implicit motor learning (see Fig. 3, right graph) in the per-protocol analysis. In addition, the study was probably underpowered due to this deviation.

Second, the use of the 10MWT as the primary outcome measure may have had implications for both the findings themselves and the interpretation in terms of clinical meaningfulness. The 10MWT was chosen as the primary outcome measure due to its validity, reliability, and feasibility within clinical practice^40^ but also to allow comparison with other studies.^41^ The advantage of using walking speed as a primary outcome is the integrated result on multiple gait parameters such as step length, frequency, and stability^14–16^ and the direct relation to changes in functional scales.^42^ In contrast to our expectations, we did not detect statistically (or clinically relevant) changes between the 2 groups. Perhaps different outcome measures (eg, kinematics and kinetics of gait) could be used to detect changes in performance, but it would also require other types of research settings and designs.

Another explanation for the neutral results could originate from the way the interventions were operationalized. Contrary to earlier studies in more controlled settings and with nonfunctional tasks such as serial reaction time tasks,^36^ it seems difficult to keep the contrast between interventions equally large when including a functional task within a clinically relevant environment. In contrast to other studies,^10^^,^^11^ the exact number of rules was not predefined but tailored to the participants. For example, Lam et al^11^ used a fixed number of 8 verbal rules compared with 1 analogy. The provided number of explicit rules (explicit intervention) may have been limited because of ethical reasons, potentially resulting in a diminished contrast between groups.

Within this study, we assessed the implicit nature of the intervention by asking participants to report the number of explicit rules they learned (verbal protocol), assessing durability of performance over a longer time period and dual-task interference.^10^^,^^11^ None of these measures revealed a clear picture of the nature of the learning process. For instance, fewer rules were accumulated in the implicit compared with the explicit group, but it remains unclear whether these rules were acquired through treatments before enrolment of this study. In addition, for some participants, the tone-counting task may have been too easy, therefore not leading to dual-task interference, whereas for other people the task was too difficult. Due to this large variation in performance on the cognitive (tone counting) dual task, it was not possible to further legitimately interpret these results.

Finally, on average both groups slightly improved their walking speed after the intervention (+0.08 m/s in the implicit group and + 0.06 m/s in the explicit group), exceeding the threshold for clinical relevant change of >0.06 m/s for within-group differences as established by Perera et al.^43^ It might be that using implicit or explicit motor learning does not make a (clinically relevant) difference for the results of walking rehabilitation within the included target group and setting of this trial. It is remarkable that the detected improvement (in both groups) remained relatively stable at the follow-up test. This finding might be seen as a form of retention and indicates that motor learning occurred rather than just a temporal improvement in motor performance.

### Future Research

The design of this RCT was carefully prepared by research into underlying theories,^44^ feasibility, and piloting testing of implicit motor learning.^12^^,^^13^ Applying the RCT in its cleanest form in clinical settings^45^ and with complex interventions was challenging because we needed to balance between external validity (generalizability of the results for daily practice) and internal validity (standardization and reliability of the results). Other designs may be considered to evaluate the effectiveness of long-term, highly individualized, and complex interventions^44^ as needed in the field of motor learning. Two recent studies suggest that tailoring motor learning interventions towards patient characteristics and preferences might be important, promoting more pragmatic trials.^9^^,^^46^ The interventions may also be applicable for people with more severe cognitive impairments (MoCA ≤ 21) as equal trends in performance were found within this subgroup. A logical next step would be to assess which patient characteristics influence motor learning interventions and how these factors influence the learning process. Therefore, cohort studies in which all potential influencing factors (eg, activity dependent plasticity, cognition, or individual preferences) are measured over time and therapists document the used motor learning approach in detail might be an interesting alternative to consider.

To gain more insight into the gait mechanisms and functional effects when applying implicit motor learning, future studies may consider combining upcoming instruments for quantitative gait analysis that can be performed outside laboratory settings (eg, use of wearable sensors)^47^^,^^48^ with patient-specific outcome measures that can detect functional relevant changes within individualized goals (eg, Patient Specific Functional Scale).^49^^,^^50^

### Clinical Message

In this study, no overall benefits of implicit motor learning over explicit motor learning for improving walking performance in people after stroke in the chronic phase of recovery were found. The treatment effects in this study may have been diluted by “noise” accompanied with research within real-life settings, complex tasks, and a representative sample of the target population. For tailored motor learning approaches, more insight is needed on the patient characteristics and preferences that influence the process of motor learning. While awaiting further results, therapists may consider both motor learning approaches to facilitate walking speed within the stroke population.

## Supplementary Material

Supplementary_data_Table_1_pzab017

Supplementary_data_Table_2_pzab017

## Clinical Trial Registration

The trial was registered at the Netherlands Trial Registry (NL6133).
