# Supplementary material for: Implicit and Explicit Motor Learning Interventions Have Similar Effects on Walking Speed in People After Stroke: A Randomized Controlled Trial
Source: Phys Ther. 2021 Jan 22;101(5):pzab017. doi: 10.1093/ptj/pzab017 (PMC8101354; doi:10.1093/ptj/pzab017)
Supplement: Supplementary_data_Table_1_pzab017 [file supplementary_data_table_1_pzab017.docx]

**Supplementary data**

**Table 1**. Mean (SD) of groups, mean (SD) difference within groups, and estimated mean (95% CI) difference between groups as established with linear mixed model.

| Outcome | Groups | | | | | | | |  | Within group difference | | | | |  | Estimated between-group difference | | | |
| --- | --- | --- | --- | --- | --- | --- | --- | --- | --- | --- | --- | --- | --- | --- | --- | --- | --- | --- | --- |
|  | Week 0  (baseline) | |  | Week 4  (post intervention) | |  | Week 8  (1-month follow-up) | |  | Week 4 minus Week 0 | |  | Week 8 minus Week 0 | |  | Week 4 minus Week 0 |  | Week 8 minus Week 0 | |
|  | Implicit (n = 5) | Explicit (n = 10) |  | Implicit (n = 5) | Explicit (n = 10) |  | Implicit (n = 5) | Explicit (n = 9) |  | Implicit | Explicit |  | Implicit | Explicit |  | Implicit-Explicit |  | Implicit-Explicit |  |
| 10 MWT *(m/s)* *performance for people scoring MoCA ≤ 21 (n=15)* | 0.47 (0.32) | 0.73 (0.30) |  | 0.51 (0.28) | 0.80 (0.31) |  | 0.51 (0.37) | 0.74 (0.32) |  | 0.04 (0.06) | 0.07 (0.12) |  | 0.05 (0.07) | 0.03 (0.17) |  | 0.04 (-0.08 to 0.17) |  | -0.02 (-0.19 to 0.15) |  |
|  | Implicit (n = 33) | Explicit (n = 31) |  | Implicit (n = 29) | Explicit (n = 28) |  | Implicit (n = 30) | Explicit (n = 27) |  | Implicit | Explicit |  | Implicit | Explicit |  | Implicit-Explicit |  | Implicit-Explicit |  |
| 10 MWT *(m/s)*  *performance for people scoring MoCA > 21 (n=64)* | 0.74 (0.37) | 0.69 (0.28) |  | 0.80 (0.37) | 0.75 (0.35) |  | 0.83 (0.39) | 0.76 (0.36) |  | 0.05 (0.14) | 0.07 (0.13) |  | 0.09 (0.15) | 0.08 (0.14) |  | 0.02 (-0.05 to 0.09) |  | -0.01 (-0.09 to 0.06) |  |

Within group differences was calculated pairwise, missing cases were excluded. Small anomalies in subtraction are due to the effects of rounding.

10MWT = 10-Meter Walk Test, MoCA = Montreal Cognitive Assessment
