# Supplementary material for: Implicit and Explicit Motor Learning Interventions Have Similar Effects on Walking Speed in People After Stroke: A Randomized Controlled Trial
Source: Phys Ther. 2021 Jan 22;101(5):pzab017. doi: 10.1093/ptj/pzab017 (PMC8101354; doi:10.1093/ptj/pzab017)
Supplement: Supplementary_data_Table_2_pzab017 [file supplementary_data_table_2_pzab017.docx]

| **Outcome** | Groups | | | | | | | |  | **Within group difference** | | | | |  | **Estimated between group difference** | | | |
| --- | --- | --- | --- | --- | --- | --- | --- | --- | --- | --- | --- | --- | --- | --- | --- | --- | --- | --- | --- |
|  | Week 0  (baseline) | |  | Week 4  (post intervention) | |  | Week 8  (1-month follow-up) | |  | Week 4 minus Week 0 | |  | Week 8 minus Week 0 | |  | Week 4 minus Week 0 |  | Week 8 minus Week 0 |  |
|  | Implicit (n = 28) | Explicit (n = 32) |  | Implicit (n = 27) | Explicit (n = 32) |  | Implicit (n = 28) | Explicit (n = 31) |  | Implicit | Explicit |  | Implicit | Explicit |  | Implicit-Explicit |  | Implicit-Explicit |  |
| 10 MWT *(m/s)* | 0.58 (0.30) | 0.64 (0.27) |  | 0.66 (0.34) | 0.72 (0.33) |  | 0.69 (0.38) | 0.69 (0.33) |  | 0.09 (0.11) | 0.08 (0.13) |  | 0.11 (0.14) | 0.06 (0.15) |  | -0.01 (-0.07 to 0.05) |  | -0.06 (-0.13 to 0.02) |  |
| DTE motor task (%) | -3.4 (25.1) | -7.8 (17.6) |  | 11.8 (34.7) | 6.3 (40.1) |  | 8.4 (36.3) | -5.6 (24.2) |  | 13.5 (32.4) | 8.4 (22.2) |  | 12.1 (32.2) | 2.5 (25.0) |  | -0.97 (-18.16 to 16.21) |  | -12.89 (-27.56 to 1.60) |  |
| DTE cognitive task (%) | -8.3 (22.2) | -14.6 (23.8) |  | -5.8 (23.4) | -8.2 (35.5) |  | -3.8 (23.8) | 1.0 (35.5) |  | 3.2 (33.1) | 1.6 (30.1) |  | 5.1 (31.3) | 10.0 (23.2) |  | -7.35 (-12.48 to 27.19) |  | 23.56 (-2.04 to 49.16) |  |
| mDGI (0 to 64) | 32.4 (14.2) | 31.8 (12.4) |  | 35.6 (14.4) | 34.6 (13.5) |  | 35.5 (16.5) | 34.1 (14.1) |  | 4.0 (5.0) | 2.9 (4.1) |  | 4.2 (5.6) | 2.6 (4.8) |  | -0.73 (-3.08 to 1.62) |  | -1.46 (-4.11 to 1.18) |  |
| MSRS (0 to 10) | 4.9 (2.7) | 5.0 (2.8) |  | 5.2 (2.7) | 4.2 (2.2) |  | 4.9 (2.5) | 4.6 (2.6) |  | 0.3 (2.1) | -0.8 (2.6) |  | 0.0 (2.3) | -0.3 (2.5) |  | -1.04 (-2.07 to -0.01) |  | -0.36 (-1.45 to 0.73) |  |
| SAQOL-39 (1 to 195) | 148 (23) | 143 (25) |  | - | - |  | 157 (22) | 148 (29) |  | - | - |  | 9 (16) | 5 (18) |  | - |  | -5.34 (-14.14 to 3.46) |  |
| Within group differences was calculated pairwise, missing cases were excluded. Small anomalies in subtraction are due to the effects of rounding.  10MWT = 10-Meter Walk Test, DT = Dual Task, mDGI = modified Dynamic Gait Index, SAQOL-39 = Stroke and Aphasia Quality of Life Scale-39 | | | | | | | | | | | | | | | | | | |  |

**Supplementary data**

**Table 2**. Mean (SD) of groups, mean (SD) difference within groups, and estimated mean (95% CI) difference between groups as established with linear mixed model.
